# Modeling the potential global distribution of the Egyptian cotton leafworm, *Spodoptera littoralis* under climate change

**DOI:** 10.1038/s41598-023-44441-8

**Published:** 2023-10-12

**Authors:** Sara M. ElShahed, Zahia K. Mostafa, Marwa H. Radwan, Eslam M. Hosni

**Affiliations:** Department of Entomology, Faculty of Science, Ain Shams University, Abbassia, 11566 Cairo Egypt

**Keywords:** Ecology, Biogeography, Climate-change ecology, Invasive species

## Abstract

The Egyptian cotton leafworm, *Spodoptera littoralis* is a highly invasive insect pest that causes extensive damage to many of the primary food crops. Considering the recent challenges facing global food production including climate change, knowledge about the invasive potential of this pest is essential. In this study, the maximum entropy model (MaxEnt) was used to predict the current global spatial distribution of the pest and the future distribution using two representative concentration pathways (RCPs) 2.6 and 8.5 in 2050 and 2070. High AUC and TSS values indicated model accuracy and high performance. Response curves showed that the optimal temperature for the *S.* *littoralis* is between 10 and 28 °C. The pest is currently found in Africa and is widely distributed across the Middle East and throughout Southern Europe. MaxEnt results revealed that the insect will shift towards Northern Europe and the Americas. Further, China was seen to have a suitable climate. We also extrapolated the impact of these results on major producing countries and how this affects trade flow, which help decision makers to take the invasiveness of such destructive pest into their account.

## Introduction

Global food production is severely threatened. It is estimated that the world’s population will climb to 9.8 billion by 2050 and in order to meet the increased food demand, production must increase by 70%^1^. Along with population growth, the Russia-Ukraine conflict is yet another problem facing food security^2^. Ukraine and Russia are considered the world’s breadbasket, supplying 30% and 20% of global wheat and maize exports as well as 80% of global exports of sunflower seed products^2,3^. This will have a drastic impact on nations highly dependent on imports from these two countries^4,5^.

Furthermore, agriculture is extremely reliant on climate and weather conditions to produce food crops and thus, global warming heavily impacts agricultural production^6–8^. Agriculture is predicted to decline in the tropics more than temperate regions; however, warming past crop thresholds will cause a decline in the temperate zone as well^8^. Climate change will also affect the physiology, distribution, phenology, and adaptation of animals and insects including insect pests^9–11^. New ecological niches are formed with optimal environmental factors for insects which allows them to expand their geographical range and shift to new regions^7,9,10^.

The Egyptian cotton leafworm, *Spodoptera littoralis*, is a major agricultural pest^12,13^. Its polyphagous larva can consume a wide range of economically important crops belonging to more than 40 families^12^. It has a wide range of host plants including wheat, maize, rice, sugarcane, soybeans, cotton, fruits, vegetables, ornamentals, orchards, castor oil trees, and many more^12–14^. Larvae mainly feed on the leaves stripping them completely except for the larger veins^14^. For that reason, the Egyptian cotton leafworm is considered to be one of the most significant cotton pests^13^. Cotton defoliation of 20 to 70% of the leaf area can cause a 50% reduction in yield^12^. This heavily affects major cotton producing countries and leads to significant economic losses^15,16^. In addition to destroying leaves, larvae were found to bore into the fruits of tomatoes, peppers, apples, and grapes making them unfit for consumption^12–14^. EPPO (European and Mediterranean Plant Protection Organization) has listed *S.littoralis* as an A2 quarantine pest^12^.

At present, the Egyptian Cotton Leafworm is found in tropical and subtropical areas^12,13^. It is native to Africa and is widely distributed across the Middle East and throughout Southern Europe. Transient populations may manifest in Northern Europe but are limited because *S.littoralis* is a non-diapausing insect that is unable to survive in low temperatures^12^. Hence, monitoring the distribution of pests is very important as invasive species are expected to proliferate more in temperate regions than in the tropics after the global temperature rise^7,17^.

Different Ecological Niche Models (ENMs) are used to determine the association between environmental conditions and species’ distribution using Geographic Information System (GIS) techniques^18^. They are becoming valuable tools for monitoring and forecasting the distribution of insect pests and their potential establishment in new regions^19^. The Maximum Entropy Model (MaxEnt) is a popular correlative model that has been successfully used to predict the effects of climate change on the distribution of numerous insect species^20^. The species distribution model has demonstrated high-performance accuracy in forecasting the future possibility of the establishment of invasive species and economically significant pests in specific locations^21–23^ and on a global scale^20,24,25^.

To our best knowledge, no ENM has been made for *S.littoralis* on a global scale. The aim of this study is to predict the global current and future distribution of the Egyptian Cotton Leafworm using MaxEnt. The results serve as a warning for countries susceptible to the invasion of *S.littoralis* as a result of the climatic shifts. We also highlight the main agricultural producing and exporting countries and how they are affected.

## Results

### Modeling performance

The MaxEnt model for the Egyptian Cotton Leafworm has a high AUC value of 0.93 (Fig. S1) indicating its significance as the AUC value tends to be higher with good modeling outputs^25^. A TSS value of 0.9 further confirmed the efficiency of the model. Values greater than 0.5 are valid.

### Contribution of bioclimatic variables

**Figure 1:**
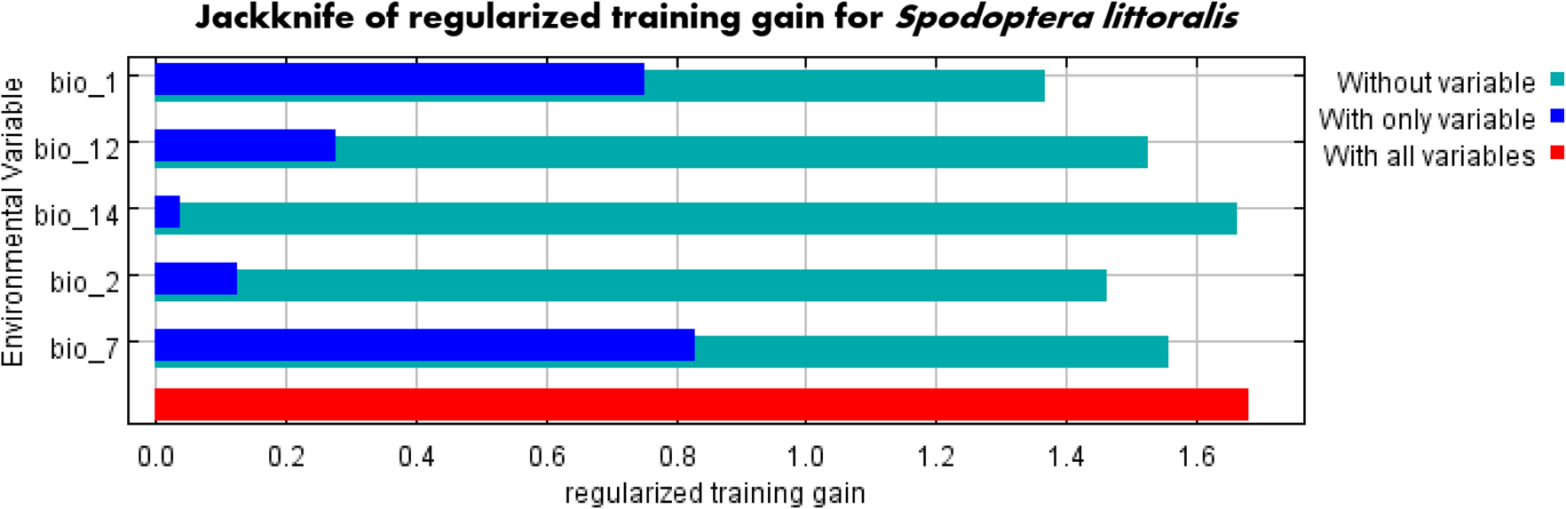
Jackknife test showing the most important variables.

The jackknife test illustrated the contribution percentages of the five most significant bioclimatic variables (Fig. 1, Table 1). The top two variables are temperature-related with Bio7 being the Temperature annual range (41.8%) and Bio1 accounting for the Annual mean temperature (32.4%). Annual precipitation (Bio12) came in third with a percentage of 10.9%. According to the response curves of the important variables, the favorable mean temperature for *S.littoralis* ranges between 10 and 28 °C (Fig. S2).

**Table 1:** Relative percentages of bioclimatic variables used in Maxent to model the current and future habitat suitability of the Egyptian cotton leafworm, *Spodoptera littoralis.*

Bioclimatic variables	Description	Contribution percentage (%)
Bio 7	Temperature annual range	41.8
Bio 1	Annual mean temperature	32.4
Bio 12	Annual precipitation	10.9
Bio 2	Mean diurnal range (mean of monthly max temp − min temp)	10.2
Bio 14	Precipitation of driest month	4.6

### Predicted current potential distribution of the Egyptian cotton leafworm

**Figure 2:**
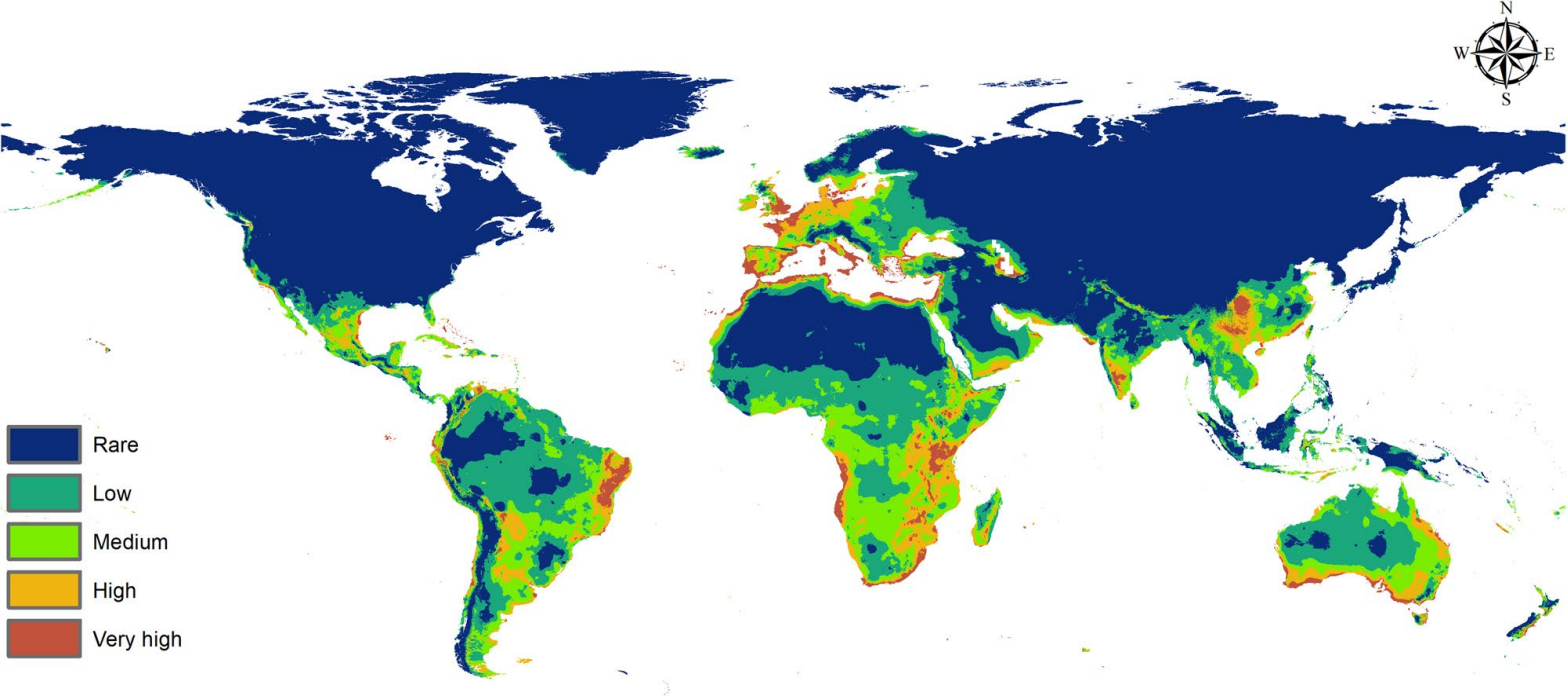
Current potential distribution of *Spodoptera littoralis* (MaxEnt v. 3.4.1 and ArcGIS v. 10.3).

The current model produced by MaxEnt predicts habitat suitability of the Egyptian Cotton Leafworm beyond its natural distribution in Africa, Europe and Asia (Fig. 2). In Africa, *S. littoralis* is concentrated in the south, including south Africa, Namibia, Zimbabwe, Mozambique and Tanzania. The pest is also found in Congo, Cameroon, Nigeria, Ghana, Gambia, Ethiopia, Kenya, Northern Egypt, Tunisia, Morocco and Madagascar. The MaxEnt model shows very high risk of the pest being established in Angola, Northern Libya, Algeria, Western Sahara along with medium to low risk of establishment in most of the continent (Fig. 2). Further, in different continents, *S. littoralis* could occupy many ecozones around the world. It is prominent that the pest is concentrated in the south which lies within the subtropical and warm temperate regions. Notably, the MaxEnt model predictions of very high suitability is found in countries in the subtropical zones, while medium to low predictions are found in the tropical regions.

**Figure 3:**
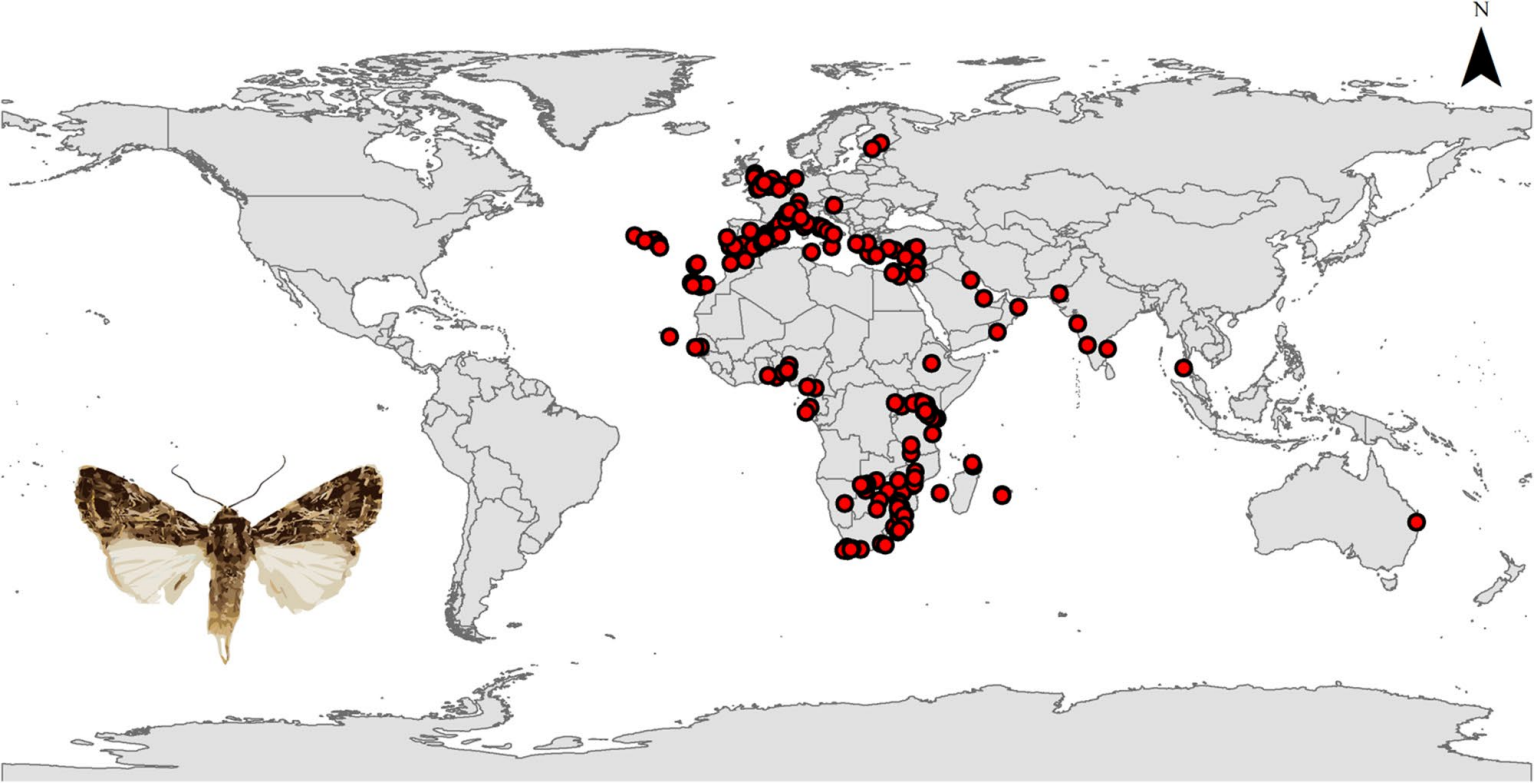
Occurrence records of *Spodoptera littoralis* used in modeling the species distribution (ArcGIS v. 10.3).

In Europe, we find the pest naturally present in the warm temperate zones in the southern parts including Spain, France, Italy, Austria, Albania, Greece, and Serbia. It is also found in the UK and in Finland (Fig. 3). The current prediction estimates very high to high habitat suitability throughout northwestern Europe and medium to low suitability in eastern European countries which lie in the cool temperate domain. Further, the model forecasts high risk in Germany, Ireland and south of Sweden (Fig. 2).

In Asia, occurrence points are seen in warm temperate to subtropical zones found in Lebanon, Syria, Iraq, Turkey, Jordan, Bahrain, Oman, Pakistan. Further, it is found in the south of India where it is in part subtropical and Thailand which lies within the tropical region (Fig. 3). Our model predicts very high risk in the warm temperate zone in China in addition to a high risk in southern Iran and in the subtropical region of southern Yemen. On the other hand, it shows high to medium suitability in Indonesia and medium to low risk in Saudi Arabia (Fig. 2).

There are no occurrence records for the Egyptian Cotton Leafworm in the Americas (Fig. 3). In North America, the model shows very high habitat suitability in the east of Mexico which is considered subtropical, western U.S. which lies in the warm temperate region, the Bahamas and south western Canada. Moreover, it reveals high to medium risk in Cuba, Jamaica and the Dominican Republic and medium risk in Alaska which lies within the cool temperate domain (Fig. 2). As for South America, the model forecasts very high suitability in eastern Brazil, which is subtropical in nature, Ecuador, Colombia, north eastern Venezuela, Peru and Chile that are subtropical to warm temperate in nature. Additionally, high to medium risk is shown in Bolivia (tropical to subtropical) and eastern Argentina in the warm temperate region (Fig. 2). Finally, in regards to Australia, the current prediction model shows very high risk of establishment in the southern coasts that lie within the warm temperate zone and also in New Zealand in the cool temperate zone (Fig. 2).

### Predicted future distribution of the Egyptian cotton leafworm

**Figure 4:**
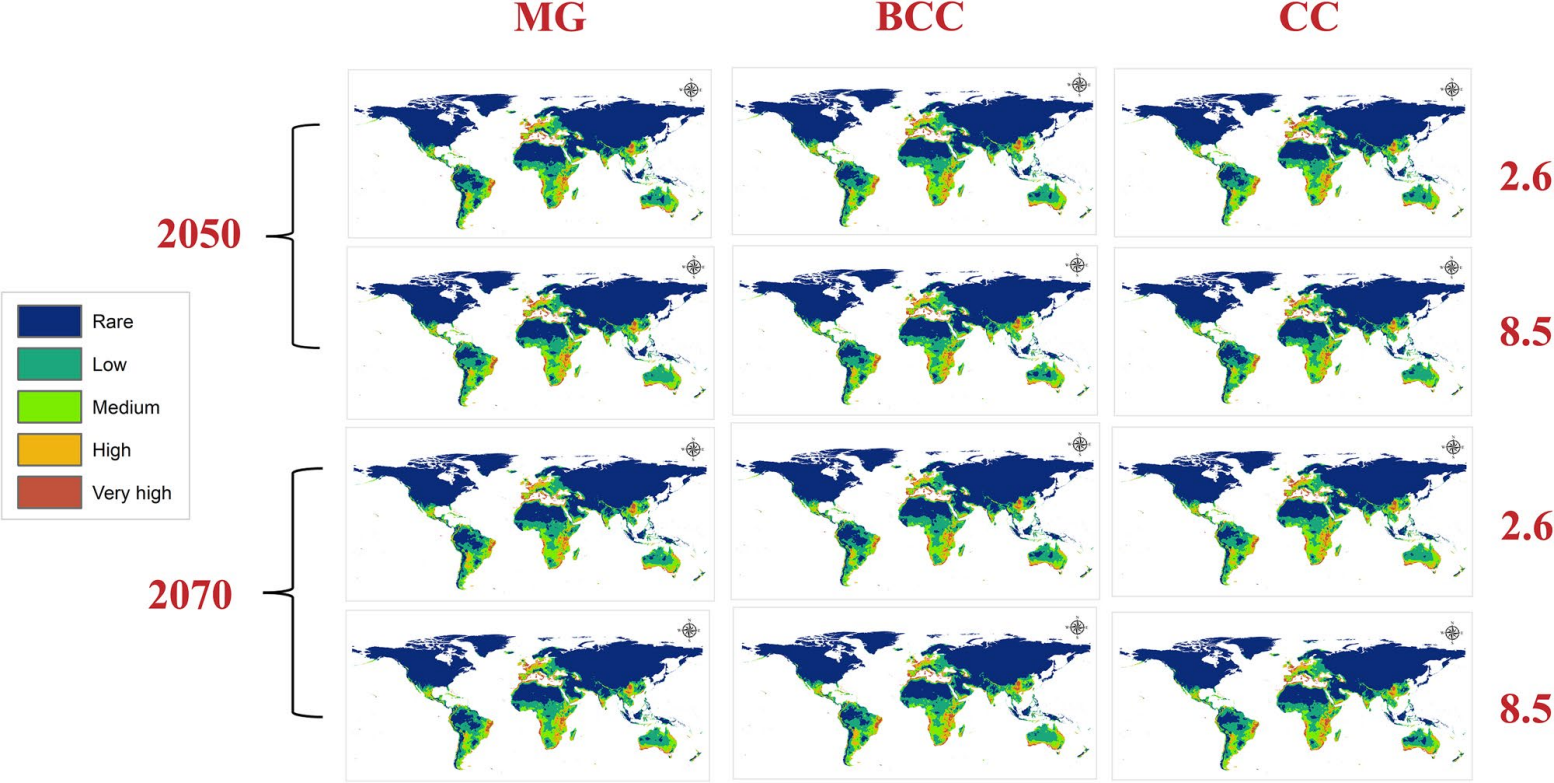
Predicted future distribution of *Spodoptera littoralis* under the RCPs 2.6 and 8.5 for three GCMs (MaxEnt v. 3.4.1 and ArcGIS v. 10.3).

**Figure 5:**
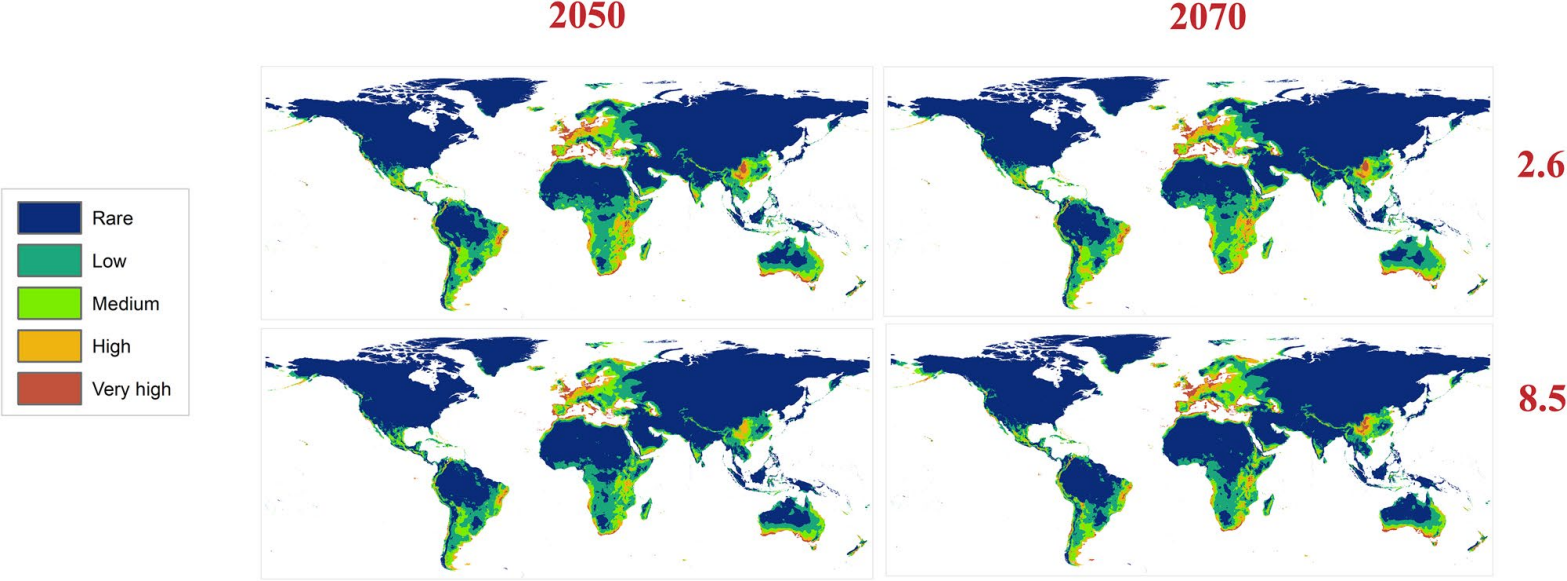
Maps showing the mean of the three GCMs using the RCPs 2.6 and 8.5 for the two time periods 2050 and 2070. MG: Meteorological Research Institute (MRI-CGCM3), BCC: Beijing Climate Center (BCC-CSM 1_1) and CC: National Center for Atmospheric Research (CCSM4) (MaxEnt v. 3.4.1 and ArcGIS v. 10.3).

**Figure 6:**
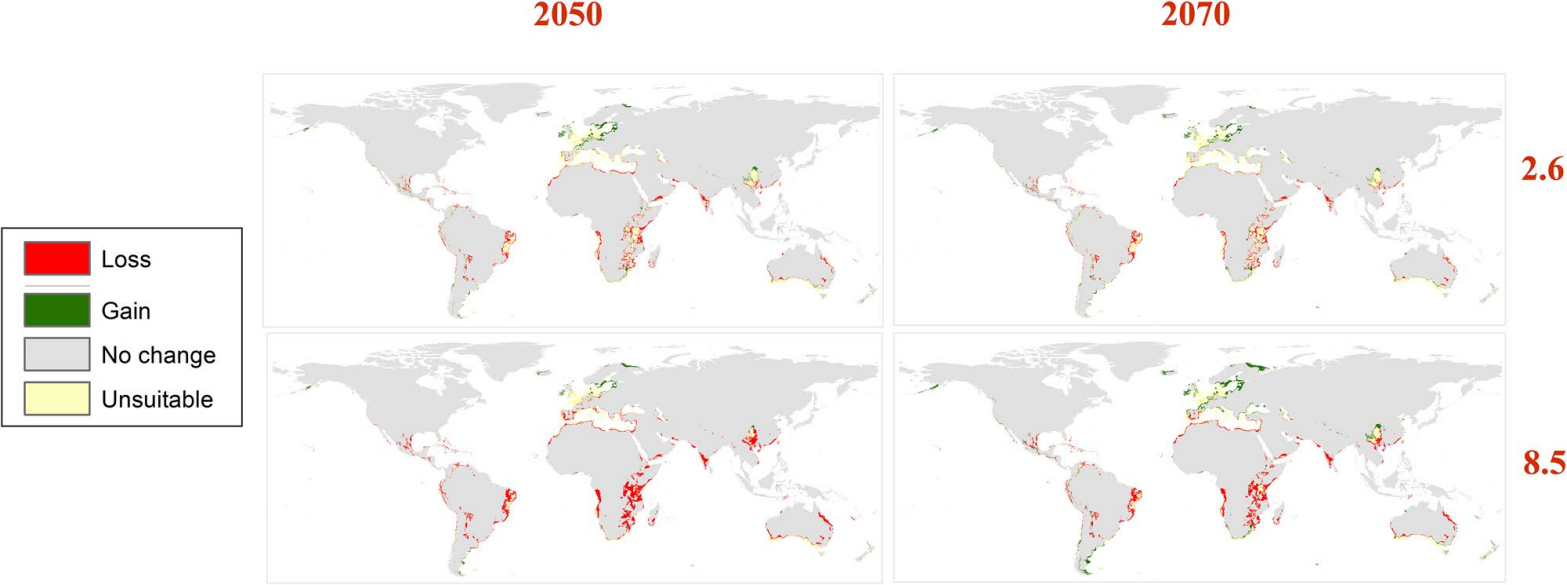
Maps showing the loss and gain in habitat suitability of *Spodoptera littoralis* using the RCPs 2.6 and 8.5 for the two time periods 2050 and 2070 (MaxEnt v. 3.4.1 and ArcGIS v. 10.3).

Three GCMs were used to assess the future distribution of the Egyptian Cotton Leafworm during 2050 and 2070 using RCPs 2.6 and 8.5 (Fig. 4). The mean risk maps of the three GCMs for both RCPs in the two time periods summarize the changes in the habitat suitability of *S. littoralis* (Fig. 5). Throughout the two RCPs (2.6 and 8.5), noticeable loss in habitat suitability is shown in Africa due to the extreme temperature elevation specifically in the tropical regions (Fig. 6).

Further, In Europe, a clear shift in the natural distribution of the pest from the south to northern European countries is depicted towards the cool temperate zone (Fig. 6). In Asia, a loss in habitat suitability is seen in countries where the pest is currently distributed in the tropics and subtropics. Moreover, a significant gain is found in China within the warm temperate region that is most visible in the RCP 8.5 2070 scenario (Fig. 6). Most countries in the Americas become less suitable for *S.littoralis* excluding Alaska (cool temperate), western U.S. (warm temperate), southeastern Argentina (warm temperate) and southwestern Chile (cool temperate), where a considerable gain is apparent (Fig. 6). As for Australia, some gain is found in the southeast and in New Zealand (Fig. 6).

## Discussion

The second sustainable development goal (SDG2) is to end global hunger, food insecurity and malnutrition by 2030^2^. According to the Food and Agriculture Organization (FAO), in 2021, between 702 and 828 million people in the world were undernourished^34^. Governments must intervene to resolve this situation and one way is to enhance agricultural production to meet the increased demand^34^. Fiscal support can target agricultural research and development in addition to inspection and control of agricultural products, diseases and pests to establish product safety according to regulations^34^. Moreover, international agricultural trade is crucial for improving food system efficiency and ensuring food security in some regions^16^. Implementation of an open trade policy increases agricultural productivity and affects food availability worldwide^35^. We cannot attain food security solely through trade policy; however, it was shown that opening trade in some regions in India led to a significant decline in famines^36^.

Half of the global production of primary crops in 2020 was attributed to four particular crops: sugar cane (20%), maize (12%), wheat and rice (8% each)^37^. Primary food crops are susceptible to infestations by *S. littoralis*, one of the most damaging agricultural insect pests^12,38^. Due to its polyphagous nature, it is extremely difficult to control and subsequently, impossible to eradicate once established^14^. Hence, monitoring its distribution regularly is a necessity to prevent it from invading and shifting to new regions under climate change. Insects are very sensitive to temperature switches and their metabolism can double with a 10 °C increase^7^. According to our MaxEnt model, temperature serves as the most significant variable affecting *S. littoralis*. Temperature annual range (bio7) and annual mean temperature (bio1) had the highest contribution percentages of 41.8% and 32.4%, respectively. Also, the response curve indicated that the optimal temperature for the Egyptian Cotton Leafworm is between 10 and 28 °C. This is significant because any rise in temperature above that range renders the environment unsuitable for the insect pest and this is apparent in the resultant models. Furthermore, regions that were once unfit for its survival become more favorable as temperature elevates.

**Figure 7:**
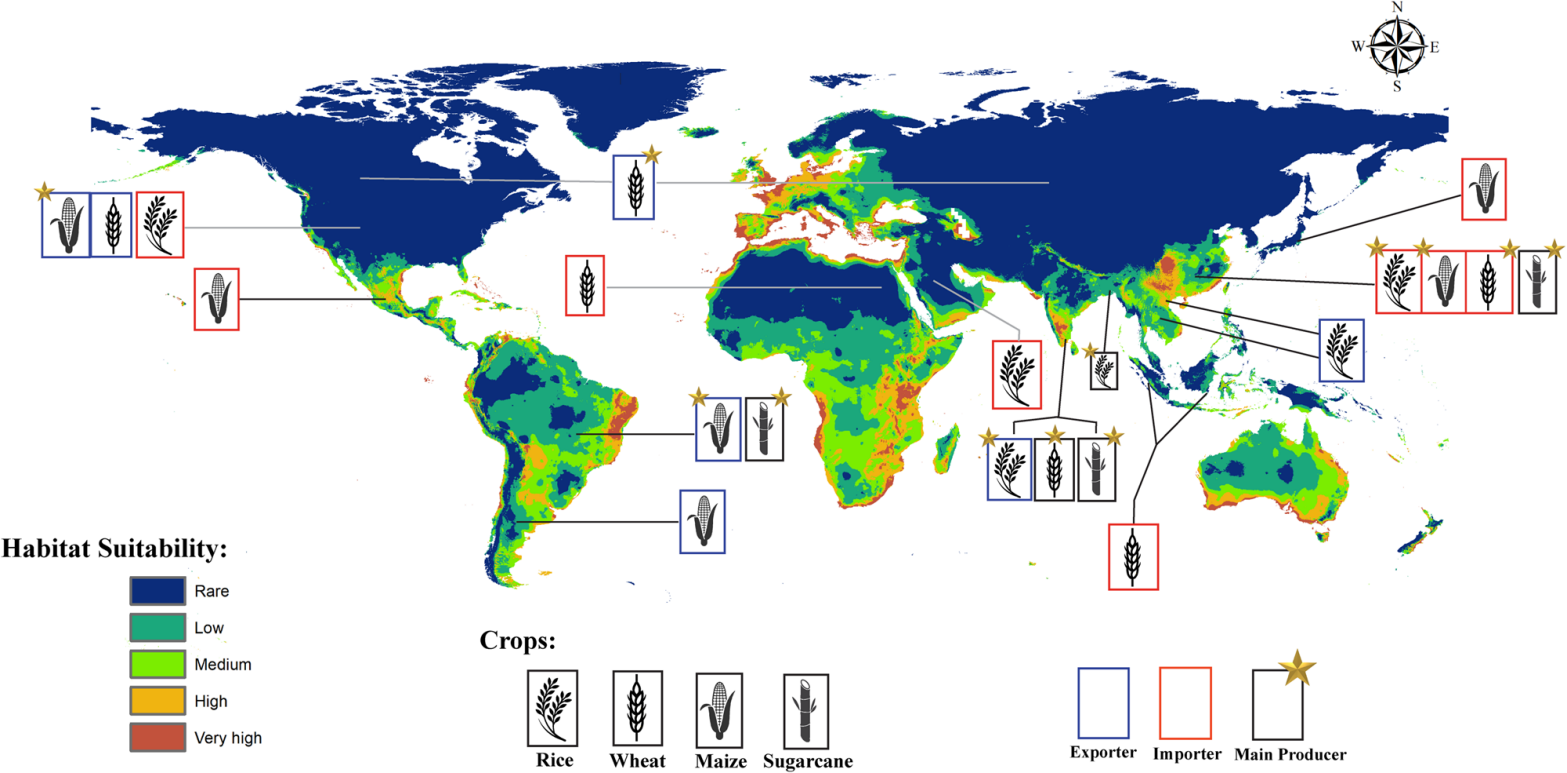
Illustration of the overlap between the current potential distribution of *S.littoralis* and the main production areas of the four major crops (rice, wheat, maize and sugarcane) while describing their status in the international trade pathways in 2020 according to the Food and Agriculture Organization (FAO).

Africa is heavily impacted by global warming despite its minimum contribution of greenhouse gas (GHG) emissions to the atmosphere^40,41^. The intergovernmental panel on climate change (IPCC) projects an increase of 3 to 6 °C across Africa by the end of the decade^40^. The effect of climate change on Africa is evident in our models. Currently, *S. littoralis* is concentrated in southern Africa and in some areas of the middle west. The current prediction estimates a very high risk of habitat suitability throughout the northern coasts and medium to low risk in most of the continent. Contrastingly, the future prediction shows a clear loss of habitat suitability in 2050 and 2070 in the two RCPs (2.6 and 8.5). The loss is seen in all regions predicted by the current model to be of high risk except for a part in South Africa that shows a gain in the high emissions scenario (RCP 8.5). Further, in the low mitigation scenarios (RCP 2.6), a yellow color is visible in South Africa and Tanzania indicating habitat unsuitability. Overall, *S. littoralis* is not considered to be a threat to Africa in the future, however, the temperature elevation across the continent is alarming. Kotir 2011 concluded that increasing temperatures in Sub-Saharan Africa will negatively impact crop production and increase hunger risk^42^. In 2020, Africa was a main importer of all food groups except for fish, fruit and vegetables^37^. In that same year, Egypt was a main importer of wheat^39^ (Fig. 7). Additionally, the Russia-Ukraine conflict heavily affects North Africa as several countries import more than 50% of their cereals from Ukraine and Russia^3,5^.

As for Europe, the current prediction indicates habitat suitability ranging from very high to high across western Europe and medium to low in eastern European countries. Almost all of Europe is suitable for the Egyptian Cotton Leafworm excluding European Russia. The future models show habitat unsuitability in northern France, Netherlands, most of Germany, most of Italy, southern coasts of Greece, France, most of the UK and eastern and western coasts of Spain, which was one of the largest net exporting countries in 2020^37^. Loss can be seen in Spain and Germany. Gain is found in Denmark, a part of Germany, some regions in the UK, South of Sweden, South of Finland, Norway, Poland, southwestern Ukraine, west and southwest of European Russia, Ireland and Iceland which reaches its maximum in 2070 at RCP 8.5.

There’s a significant variation in trade flows between regions and food groups^37^. In 2020, Europe had one of the largest individual flows for fruit and vegetables, with USD 138 billion import value and USD 101 billion export value^37^. Furthermore, Europe was a net exporter of most commodity groups with cereals having an export value of USD 98.04 billion^37^. According to the Maxent model output maps, the situation in Europe is critical. Almost the entire continent is at risk for infestation by *S. littoralis* in the current prediction and while the future maps show loss in suitability in some countries, the gain is widespread in northern Europe including Ukraine, which is one of the world’s main breadbaskets^3^.

In Asia, the current model shows very high to high suitability in China and Thailand, high to medium risk in Indonesia. Additionally, it predicts a high risk in south to southwestern Iran and this agrees with the findings of Falsafi et al.^43^. The model also forecasts a high risk in Yemen. Notably, the current map shows a medium to low risk of establishment in Saudi Arabia. Future prediction illustrates a loss of suitability in both Iran and Yemen which reaches a maximum in the RCP 8.5 2070 scenario. Additionally, the future model predicts a significant gain in China. Further, temperature rise leads to a loss in suitability in India and some parts of China that were rendered of very high to high risk of establishment in the current model.

Over the next decade, the world’s crop production is predicted to increase by 18% mostly in China (30%), India (17%) and Asia and the Pacific region (14%)^16^. Asia was the leading continent in the production of rice (89%), potatoes (50%) and wheat (46%) in 2020^37^. In that same year, China was solely responsible for 25% of the world’s rice production and potatoes and 18% of the world’s wheat output^37^. It was also one of the main sugarcane producers^37,39^. It is evident that China is a major producing country, however its main focus is on domestic demand and not exportation^37^. Due to the increased demand, China is dependent on imports from other countries in addition to its own production^39^ (Fig. 7). Further, considering China’s reliance on agriculture and importation for food, the Maxent model predictions serve as an early warning. China’s current prediction shows a very high to high risk of establishment of *S.littoralis* and also, a significant gain in habitat suitability is clearly depicted in the future forecasts. According to the FAO, China was a main importer of rice, wheat and maize in 2020^39^ (Fig. 7). This places the country at a higher risk of potential pest introduction from regions where the insect is already established in the current period.

Concerning North America, there are no records of the Egyptian Cotton Leafworm in the U.S however, it has been intercepted at U.S ports of entry 65 times since 2004. It is listed as being of high invasive risk to the U.S^44^. The United States of America accounted for 31% of the world production of Maize in 2020 and it was also the largest food exporter (9%) succeeded by the Netherlands (6%) and China (5%)^37^. Our current MaxEnt model indicates very high to medium risk in western U.S. Moreover, the future model shows a gain in suitability in that same area which is California. Our findings agree with the DDRP and CLIMEX models made by Barker and Coup in 2021 for predicting the potential establishment of *S.littoralis* in the U.S^44^. A gain in suitability is found as well in Alaska and west of Mexico.

As mentioned, temperature is a significant factor influencing the distribution of *S.littoralis* and its establishment into new environments currently and in the future. Another element to take into consideration is international trade. Regions that have no current natural distribution of *S.littoralis* must abide by quarantine protocols when importing from areas where the pest is known to exist. Furthermore, countries like California that show high habitat suitability have to be stricter when handling imports from overseas (Fig. 7). In 2022, Mexico’s import value of maize and wheat from the U.S reached a total of $4.92 and $1.58 billion, respectively^45^. Consequently, if an infestation were to happen in the U.S, it could easily spread to Mexico where the current model predicts high to medium suitability of establishment. It is also important to note that the future model shows a loss in east Mexico, the Bahamas, Jamaica, Cuba, and the Dominican Republic all which had very high to medium risk in the current model.

For South America, the current model predicts very high suitability in Brazil and Colombia, where Brazil alone was responsible for 40% of the global production of sugar cane in 2020^37^. Ecuador, northeastern Venezuela, Peru and Chile were also of very high risk. The future model forecasts a loss in those areas due to temperature rise. In relation to Argentina, one of the largest food exporting countries in 2020^37^, a visible gain is seen in the southeast along with a gain in southwestern Chile. Lastly, in Australia there’s a general loss in habitat suitability with some gain in New Zealand and the southeast of the continent.

It is clear that *S.littoralis* occupies a range of ecological zones around the globe. According to the world temperature domains map by Sayre et al. 2020^46^, the Egyptian Cotton Leafworm is mostly currently found in two zones: subtropical and warm temperate. Also, it is present in a few tropical regions such as Ghana, Oman and parts of India. Further, based on the current MaxEnt model, Very high to high suitability is mainly seen in subtropical and warm temperate regions, while medium to low suitability can be found in some tropic and cold temperate domains.

On another note, diapause has never been reported for *S.littoralis* and it is known to overwinter where winters are warm^14^. The MaxEnt current and future models predict habitat suitability for the pest in temperate regions with cold temperatures below the 10◦C optimum. Concerning the future models, it could be argued that the global temperature will rise making the conditions more favorable for the insect. However, this does not explain current model results found in areas like Alaska and northern Europe. The current prediction estimates very high to high habitat suitability throughout northwestern Europe and medium to low suitability in eastern European countries which lie in the cool temperate domain. Adult moths are known to have a 1.5 km flight range for 4 h in order to oviposit and disperse on different hosts^12^.The Egyptian Cotton Leafworm has been trapped outside its range in northern Europe due to migratory flights from the southern parts or even infested importations^14^. Nevertheless, dispersal in cool areas would be limited by the moths’ short life^14^. As for Alaska, the model shows medium habitat suitability, but the pest would not survive the winters.

The resultant MaxEnt maps have several limitations. The software determines regions that have most similar conditions to the species current known occurrence points and arranges them from 0 (unsuitable) to 1 (suitable)^19^. Our model does not include future data about human population or host plant distribution nor does it consider the physiology or behavior of the insect and is solely based on climatic variables. Temperature, one of the main factors affecting abundance and distribution of species, impacts pest physiology in addition to the physiology of the host plant itself^7^. Moreover, CO2 elevation due to climate change increases the plant’s carbon to nitrogen ratio which leads to a decrease in the protein content and consequently, more damage by pests to compensate for the low food quality^47^. Biotic interactions are complex with numerous variables to consider such as crop yields, natural enemies, pests, weed, plant diseases and many others^47,48^. Although correlative models mainly depend on bioclimatic data, the occurrence records included implicitly capture processes such as biotic interactions and dispersal limitations^49,50^. This is an indication that models like MaxEnt give predictions that are closer to the realized niche, which is the actual environment occupied by the species^50^. The resultant maps are considered an important indicator of the invasive pattern of *S.littoralis* and encourage further research on this polyphagous agricultural pest and its socioeconomic effect.

## Conclusion

The Egyptian Cotton Leafworm is a polyphagous and a highly invasive insect pest that causes damage to many economically important food crops. In this study, we have successfully made an ecological niche model to assess habitat suitability for the pest currently and in the future under different scenarios of climate change. The resultant maps showed current areas with habitat suitability and other regions at risk of invasion by *S. littoralis* in the future. Considering the threats facing food security and the SDG2^2^, it is important to encourage more research on pest monitoring and especially those that are highly invasive and cause extensive damage to primary food crops like *S. littoralis*. Species distribution models could help quarantine authorities hasten control programs for such pests. Moreover, implementing more data into the predictive models, such as future data on human populations and hosts, would lead to a better understanding of the socioeconomic effect of the pest.

## Materials and methods

### Occurrence records

Occurrence data was obtained from the Global Biodiversity Information Facility database https://www.gbif.org. The Egyptian cotton leafworm is mainly found in Mediterranean and middle eastern countries along with southern Europe. Records were subjected to three filtration steps: First, removal of records without geo-referencing; second, removal of duplicated records and third, spatial rarefaction based on distance using ArcGIS (SDM toolbox: SDM tools; Universal tools—Spatially rarefy occurrence data)^20^. Then, the end total of 201 records were converted to .CSV format and used to predict the distribution of *S.littoralis* (Fig. 3).

### Environmental variables

Nineteen bioclimatic variables were downloaded from the WorldClim database https://www.worldclim.org with a spatial resolution of 2.5 arc-min at the equator. Forecast stations collected monthly temperature and rainfall readings between 1950 and 2000 to create these variables.

Concerning the current prediction bioclimatic data, only fifteen variables were converted into ASCII format by ArcGIS v 10.3. The bioclimatic covariates 8–9 and 18–19 were eliminated because of spatial irregularities which affect the resolution of the resulting layers^26,27^. We separated the correlated bioclimatic variables using Pearson’s correlation coefficient at a value equal to (|r|≥ 0.8) to remove correlation between covariates^20^ (Table S1). Multicollinearity was reduced through the function of SDM tools in ArcGIS 10.3 (universal tool; explore climate data; remove highly correlated variable)^28^. The five most significant bioclimatic variables were chosen for additional analysis.

As for the future prediction data, a corresponding set of bioclimatic layers was obtained from https://www.worldclim.org for the representative concentration pathways (RCPs) 2.6 and 8.5 for the two periods 2050 and 2070. RCPs explore possible future climate dynamics for CO2 emissions and the associated atmospheric concentration, RCP 2.6 being the lowest mitigation scenario and RCP 8.5 being the high emission scenario^20,29^. These layers were also converted to ASCII format using ArcGIS v 10.3 and applied to model the future prediction.

We used three General Circulation Models (GCMs) for each RCP (2.6 and 8.5) in both time periods of 2050 and 2070. The GCMs used are the Meteorological Research Institute (MRI-CGCM3), the National Center for Atmospheric Research (CCSM4) and the Beijing Climate Center (BCC-CSM 1_1) which are part of the IPCC’s fifth assessment report for the GCM climate estimates. We then calculated the mean future distribution for each RCP for all three GCMs in 2050 and 2070 to compare it with the predicted current distribution.

### Modeling approach

We chose MaxEnt software package v 3.4.1 to predict the current and future global distribution of *S. littoralis*^30^. In addition to its simplicity and ease of usage, MaxEnt is preferred over other software because it applies environmental variables other than climate (such as land cover, distance, and geographical factors) and evaluates their significance based on species’ occurrence points^31^. It has outperformed other softwares and thus, it is widely used to predict possible species distributions using presence-only records^20,27,32,33^.

In our MaxEnt models, 25% of the occurrence records were used for testing while the remaining 75% were used for training. The number of background points was 10,000 and the iterations were set at 500. To enhance the model’s performance, tenfold cross-validation was implemented. Regions with habitat suitability in the resultant models were put into five classes (Rare, low, medium, high and very high) using ArcGIS v.10.3 function (Classified-Symbology- Natural breaks (jenks)) in layer properties.

### Model evaluation

The resultant models were assessed using the Area Under the Curve (AUC) of the Receiver Operating Characteristics (ROCs) and the True Skill Statistics (TSS). AUC values ranged from 0 (random discrimination) to 1 (perfect discrimination). AUC values greater than 0.75 were seen as well-fitting, while those less than 0.5 were considered poor-fitting. TSS was used to further evaluate the models’ accuracy with values ranging from 0 to 1. Values close to 1 implied a good relationship between the model and the distribution, while values close to 0 implied a weak relationship.

### Supplementary Information

Supplementary Figure S1.

Supplementary Figure S2.

Supplementary Table S1.

## Data Availability

All the data are included in the manuscript.
