# Supplementary material for: Modeling the potential global distribution of the Egyptian cotton leafworm, Spodoptera littoralis under climate change
Source: Sci Rep. 2023 Oct 12;13:17314. doi: 10.1038/s41598-023-44441-8 (PMC10570271; doi:10.1038/s41598-023-44441-8)

Figure S1: The receiver operating characteristic (ROC) curve for Egyptian Cotton Leafworm *Spodoptera littoralis*

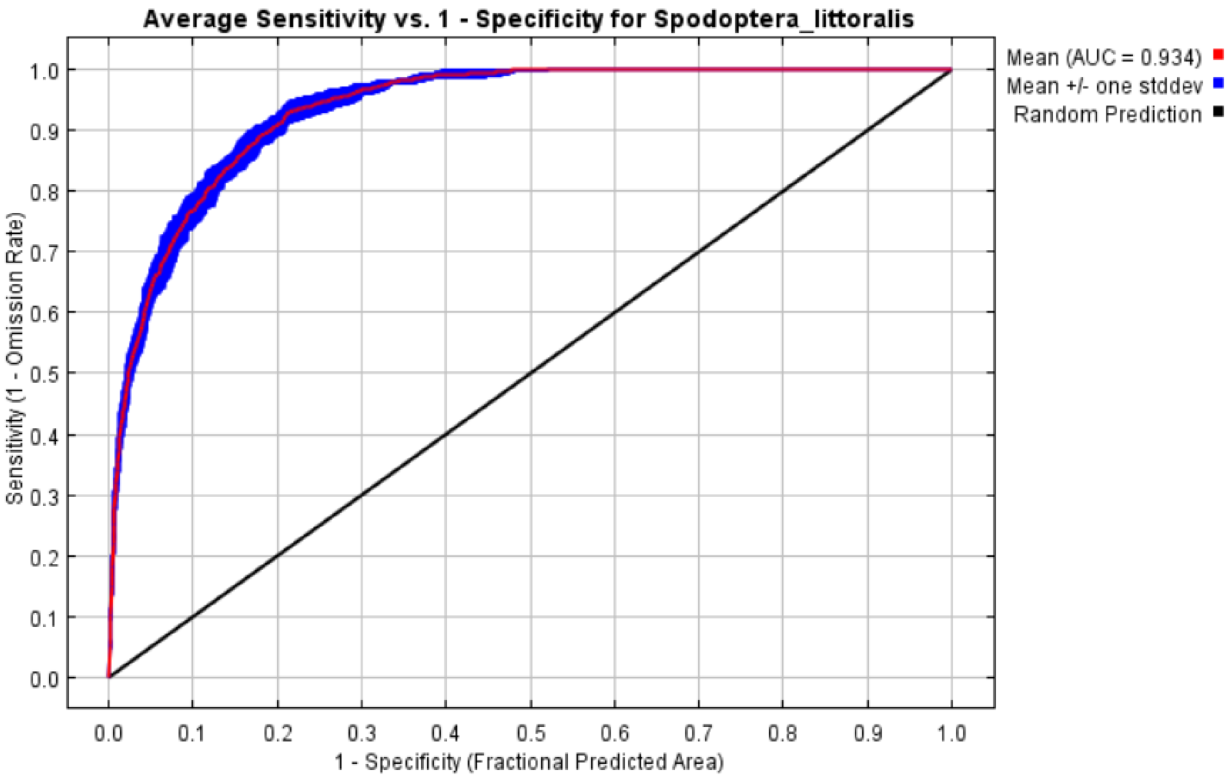

Supplement: Supplementary file 1 — Supplementary Figure S1. [file 41598_2023_44441_MOESM1_ESM.pdf]
