# Supplementary material for: Modeling the potential global distribution of the Egyptian cotton leafworm, Spodoptera littoralis under climate change
Source: Sci Rep. 2023 Oct 12;13:17314. doi: 10.1038/s41598-023-44441-8 (PMC10570271; doi:10.1038/s41598-023-44441-8)
Supplement: Supplementary file 2 — Supplementary Figure S2. [file 41598_2023_44441_MOESM2_ESM.pdf]

Figures S2: Response curves of the most relevant environmental factors affecting the distribution of the Egyptian Cotton Leafworm *Spodoptera littoralis*; the shown values are average of ten replicate runs.

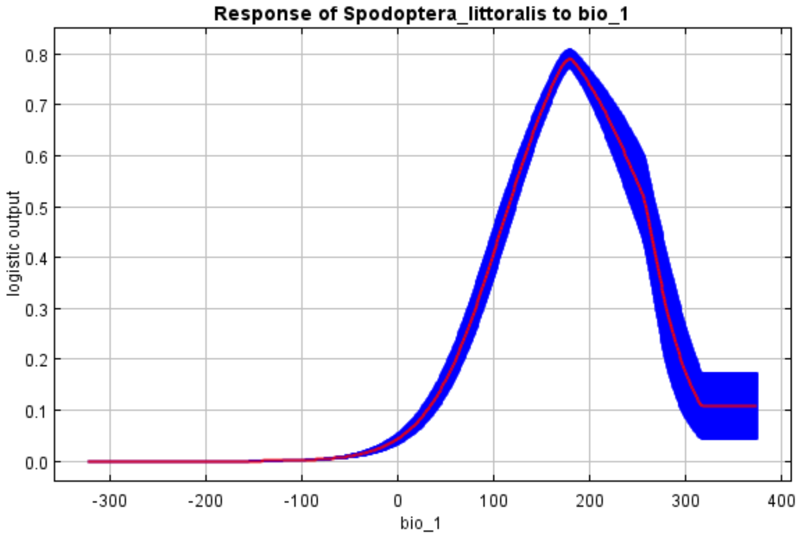

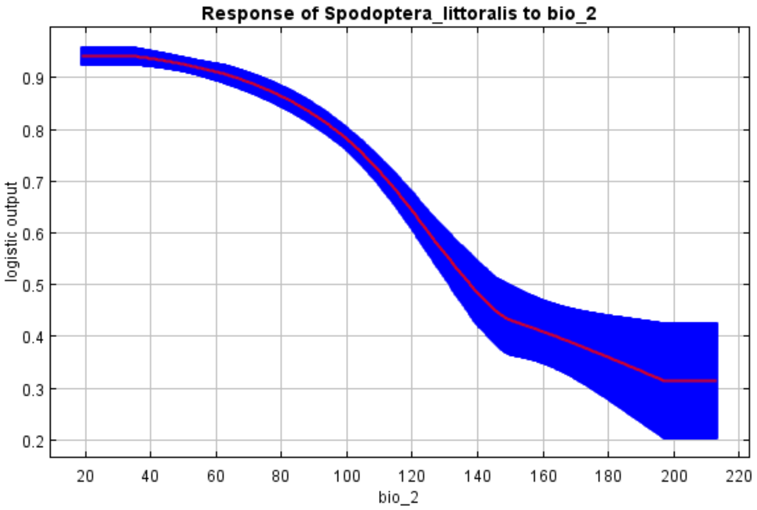

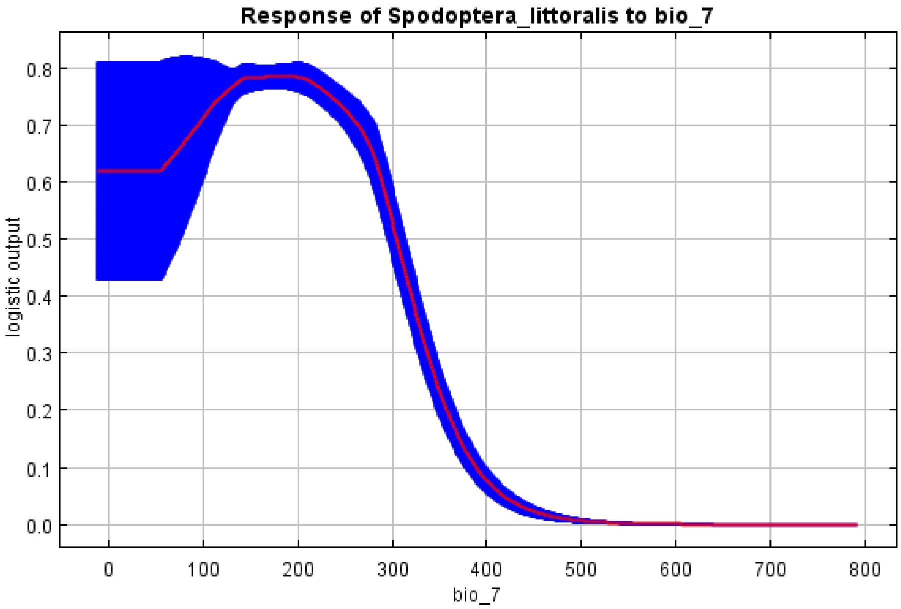
Response of Spodoptera_littoralis to bio_7
logistic output
bio_7
0.0
0.1
0.2
0.3
0.4
0.5
0.6
0.7
0.8
0
100
200
300
400
500
600
700
800

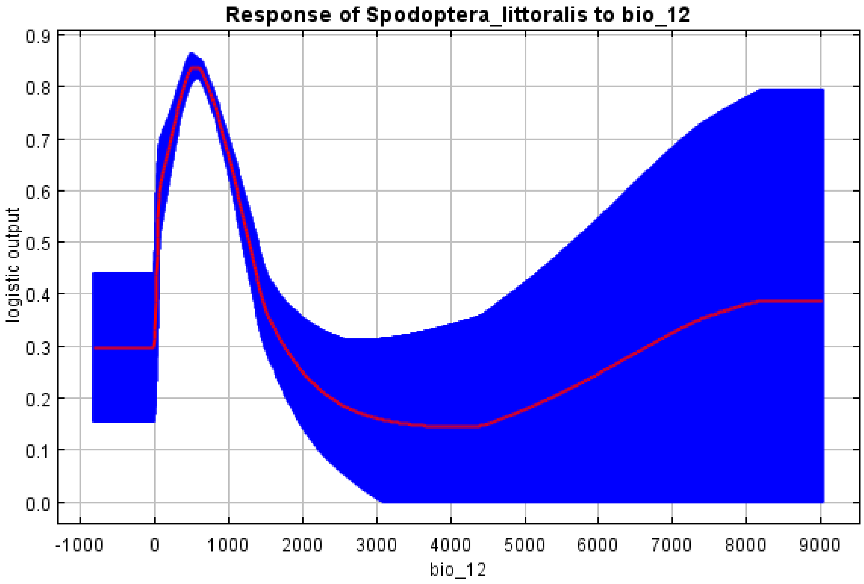
Response of Spodoptera_littoralis to bio_12
logistic output
bio_12
0.0
0.1
0.2
0.3
0.4
0.5
0.6
0.7
0.8
0.9
-1000
0
1000
2000
3000
4000
5000
6000
7000
8000
9000

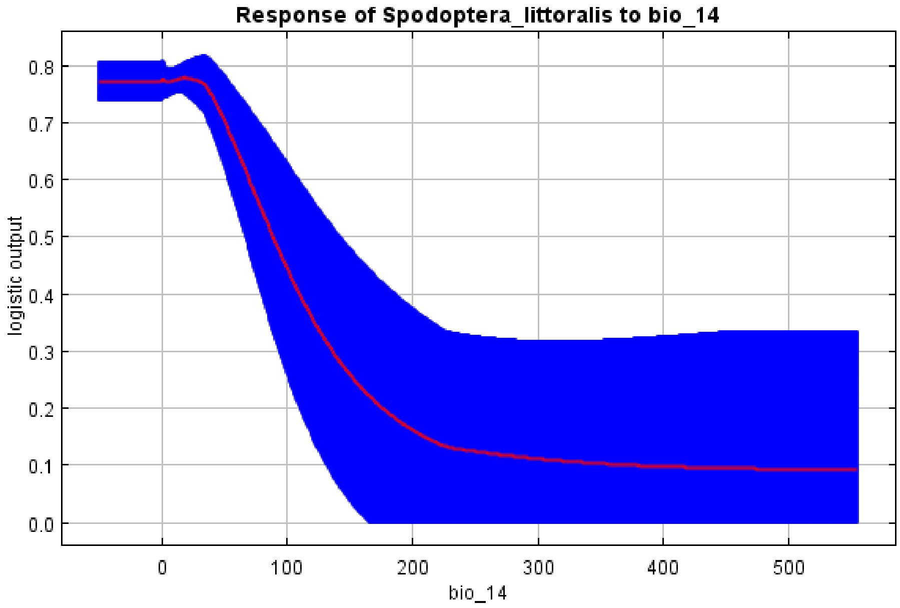
Response of Spodoptera_littoralis to bio_14
logistic output
0.8
0.7
0.6
0.5
0.4
0.3
0.2
0.1
0.0
0
100
200
300
400
500
bio_14
