# Supplementary material for: Modeling the potential global distribution of the Egyptian cotton leafworm, Spodoptera littoralis under climate change
Source: Sci Rep. 2023 Oct 12;13:17314. doi: 10.1038/s41598-023-44441-8 (PMC10570271; doi:10.1038/s41598-023-44441-8)
Supplement: Supplementary file 3 — Supplementary Table S1. [file 41598_2023_44441_MOESM3_ESM.pdf]

Table S1: Pearson's correlation coefficient test at (|r|≥ 0.8) to remove multicollinearity among the 19 bioclimatic layers.

Layer 1	Layer 2	Full ID of L	Full ID of L	Pearson's	Absolute value of R
17	18	bio_18	bio_19	0.374506	0.374506
16	18	bio_17	bio_19	0.69853	0.69853
16	17	bio_17	bio_18	0.580928	0.580928
15	18	bio_16	bio_19	0.609991	0.609991
15	17	bio_16	bio_18	0.761148	0.761148
15	16	bio_16	bio_17	0.466359	0.466359
14	18	bio_15	bio_19	0.67117	0.67117
14	17	bio_15	bio_18	0.557614	0.557614
14	16	bio_15	bio_17	0.993788	0.993788
14	15	bio_15	bio_16	0.429049	0.429049
13	18	bio_14	bio_19	0.67117	0.67117
13	17	bio_14	bio_18	0.557614	0.557614
13	16	bio_14	bio_17	0.993788	0.993788
13	15	bio_14	bio_16	0.429049	0.429049
13	14	bio_14	bio_15	1	1
12	18	bio_13	bio_19	0.584208	0.584208
12	17	bio_13	bio_18	0.742413	0.742413
12	16	bio_13	bio_17	0.429498	0.429498
12	15	bio_13	bio_16	0.992615	0.992615
12	14	bio_13	bio_15	0.392339	0.392339
12	13	bio_13	bio_14	0.392339	0.392339
11	18	bio_12	bio_19	0.753869	0.753869
11	17	bio_12	bio_18	0.796836	0.796836
11	16	bio_12	bio_17	0.743767	0.743767
11	15	bio_12	bio_16	0.922165	0.922165
11	14	bio_12	bio_15	0.70862	0.70862
11	13	bio_12	bio_14	0.70862	0.70862
11	12	bio_12	bio_13	0.896163	0.896163
10	18	bio_11	bio_19	0.315888	0.315888
10	17	bio_11	bio_18	0.265306	0.265306
10	16	bio_11	bio_17	0.15256	0.15256
10	15	bio_11	bio_16	0.504329	0.504329
10	14	bio_11	bio_15	0.123146	0.123146
10	13	bio_11	bio_14	0.123146	0.123146
10	12	bio_11	bio_13	0.511218	0.511218
10	11	bio_11	bio_12	0.4538	0.4538
9	18	bio_10	bio_19	0.11205	0.11205
9	17	bio_10	bio_18	0.100532	0.100532
9	16	bio_10	bio_17	-0.05417	0.054172
9	15	bio_10	bio_16	0.286327	0.286327
9	14	bio_10	bio_15	-0.07535	0.075351
9	13	bio_10	bio_14	-0.07535	0.075351
9	12	bio_10	bio_13	0.303114	0.303114
9	11	bio_10	bio_12	0.199822	0.199822
9	10	bio_10	bio_11	0.847842	0.847842
8	18	bio_9	bio_19	0.307043	0.307043

8	17	bio_9	bio_18	0.152563	0.152563
8	16	bio_9	bio_17	0.134592	0.134592
8	15	bio_9	bio_16	0.40789	0.40789
8	14	bio_9	bio_15	0.107479	0.107479
8	13	bio_9	bio_14	0.107479	0.107479
8	12	bio_9	bio_13	0.413302	0.413302
8	11	bio_9	bio_12	0.374582	0.374582
8	10	bio_9	bio_11	0.949983	0.949983
8	9	bio_9	bio_10	0.831251	0.831251
7	18	bio_8	bio_19	0.073949	0.073949
7	17	bio_8	bio_18	0.254073	0.254073
7	16	bio_8	bio_17	-0.04362	0.043622
7	15	bio_8	bio_16	0.352129	0.352129
7	14	bio_8	bio_15	-0.06601	0.066014
7	13	bio_8	bio_14	-0.06601	0.066014
7	12	bio_8	bio_13	0.370054	0.370054
7	11	bio_8	bio_12	0.251716	0.251716
7	10	bio_8	bio_11	0.731076	0.731076
7	9	bio_8	bio_10	0.865048	0.865048
7	8	bio_8	bio_9	0.609321	0.609321
6	18	bio_7	bio_19	-0.47528	0.475278
6	17	bio_7	bio_18	-0.38914	0.389141
6	16	bio_7	bio_17	-0.37534	0.375337
6	15	bio_7	bio_16	-0.59476	0.594762
6	14	bio_7	bio_15	-0.34565	0.345647
6	13	bio_7	bio_14	-0.34565	0.345647
6	12	bio_7	bio_13	-0.58874	0.588739
6	11	bio_7	bio_12	-0.61496	0.614965
6	10	bio_7	bio_11	-0.84693	0.846926
6	9	bio_7	bio_10	-0.45451	0.454513
6	8	bio_7	bio_9	-0.78123	0.781227
6	7	bio_7	bio_8	-0.38729	0.387287
5	18	bio_6	bio_19	0.356557	0.356557
5	17	bio_6	bio_18	0.282138	0.282138
5	16	bio_6	bio_17	0.196829	0.196829
5	15	bio_6	bio_16	0.521814	0.521814
5	14	bio_6	bio_15	0.166661	0.166661
5	13	bio_6	bio_14	0.166661	0.166661
5	12	bio_6	bio_13	0.52682	0.52682
5	11	bio_6	bio_12	0.486377	0.486377
5	10	bio_6	bio_11	0.996005	0.996005
5	9	bio_6	bio_10	0.824798	0.824798
5	8	bio_6	bio_9	0.948706	0.948706
5	7	bio_6	bio_8	0.704182	0.704182
5	6	bio_6	bio_7	-0.87585	0.87585
4	18	bio_5	bio_19	0.054833	0.054833
4	17	bio_5	bio_18	0.025942	0.025942

4	16	bio_5	bio_17	-0.12065	0.12065
4	15	bio_5	bio_16	0.214902	0.214902
4	14	bio_5	bio_15	-0.13935	0.139347
4	13	bio_5	bio_14	-0.13935	0.139347
4	12	bio_5	bio_13	0.232634	0.232634
4	11	bio_5	bio_12	0.11936	0.11936
4	10	bio_5	bio_11	0.795358	0.795358
4	9	bio_5	bio_10	0.988386	0.988386
4	8	bio_5	bio_9	0.791626	0.791626
4	7	bio_5	bio_8	0.844862	0.844862
4	6	bio_5	bio_7	-0.35843	0.358426
4	5	bio_5	bio_6	0.764447	0.764447
3	18	bio_4	bio_19	-0.40461	0.404611
3	17	bio_4	bio_18	-0.3366	0.336603
3	16	bio_4	bio_17	-0.27478	0.274785
3	15	bio_4	bio_16	-0.56867	0.568669
3	14	bio_4	bio_15	-0.24486	0.244857
3	13	bio_4	bio_14	-0.24486	0.244857
3	12	bio_4	bio_13	-0.56722	0.567221
3	11	bio_4	bio_12	-0.55374	0.553744
3	10	bio_4	bio_11	-0.92573	0.92573
3	9	bio_4	bio_10	-0.58473	0.58473
3	8	bio_4	bio_9	-0.8608	0.860803
3	7	bio_4	bio_8	-0.50138	0.501379
3	6	bio_4	bio_7	0.971165	0.971165
3	5	bio_4	bio_6	-0.93584	0.935836
3	4	bio_4	bio_5	-0.51299	0.512991
2	18	bio_3	bio_19	0.430679	0.430679
2	17	bio_3	bio_18	0.352778	0.352778
2	16	bio_3	bio_17	0.254811	0.254811
2	15	bio_3	bio_16	0.580976	0.580976
2	14	bio_3	bio_15	0.221026	0.221026
2	13	bio_3	bio_14	0.221026	0.221026
2	12	bio_3	bio_13	0.580627	0.580627
2	11	bio_3	bio_12	0.564268	0.564268
2	10	bio_3	bio_11	0.890693	0.890693
2	9	bio_3	bio_10	0.659754	0.659754
2	8	bio_3	bio_9	0.808222	0.808222
2	7	bio_3	bio_8	0.637936	0.637936
2	6	bio_3	bio_7	-0.82642	0.826419
2	5	bio_3	bio_6	0.887705	0.887705
2	4	bio_3	bio_5	0.613248	0.613248
2	3	bio_3	bio_4	-0.89144	0.891443
1	18	bio_2	bio_19	-0.24223	0.242234
1	17	bio_2	bio_18	-0.20221	0.202206
1	16	bio_2	bio_17	-0.37167	0.371668
1	15	bio_2	bio_16	-0.12436	0.124356

1	14	bio_2	bio_15	-0.37462	0.37462
1	13	bio_2	bio_14	-0.37462	0.37462
1	12	bio_2	bio_13	-0.10414	0.104141
1	11	bio_2	bio_12	-0.24488	0.244885
1	10	bio_2	bio_11	0.42557	0.42557
1	9	bio_2	bio_10	0.621295	0.621295
1	8	bio_2	bio_9	0.438617	0.438617
1	7	bio_2	bio_8	0.532916	0.532916
1	6	bio_2	bio_7	0.015384	0.015384
1	5	bio_2	bio_6	0.354887	0.354887
1	4	bio_2	bio_5	0.707079	0.707079
1	3	bio_2	bio_4	-0.2099	0.209899
1	2	bio_2	bio_3	0.864785	0.864785
0	18	bio_1	bio_19	0.248916	0.248916
0	17	bio_1	bio_18	0.223736	0.223736
0	16	bio_1	bio_17	0.082851	0.082851
0	15	bio_1	bio_16	0.444554	0.444554
0	14	bio_1	bio_15	0.055368	0.055368
0	13	bio_1	bio_14	0.055368	0.055368
0	12	bio_1	bio_13	0.455195	0.455195
0	11	bio_1	bio_12	0.378449	0.378449
0	10	bio_1	bio_11	0.979756	0.979756
0	9	bio_1	bio_10	0.93509	0.93509
0	8	bio_1	bio_9	0.938065	0.938065
0	7	bio_1	bio_8	0.8119	0.8119
0	6	bio_1	bio_7	-0.73042	0.730416
0	5	bio_1	bio_6	0.967553	0.967553
0	4	bio_1	bio_5	0.895965	0.895965
0	3	bio_1	bio_4	-0.83296	0.832961
0	2	bio_1	bio_3	0.838626	0.838626
0	1	bio_1	bio_2	0.520706	0.520706
